# A comparison of the efficacy of Chinese herbal medicines in the treatment of primary dysmenorrhea: A network meta-analysis protocol

**DOI:** 10.1097/MD.0000000000015100

**Published:** 2019-04-05

**Authors:** Li Gao, Zhuoran Xiao, Chunhua Jia, Wei Wang

**Affiliations:** School of Preclinical Medicine, Beijing University of Chinese Medicine, Beijing, China.

**Keywords:** Chinese herbal medicine, network meta-analysis, primary dysmenorrhea

## Abstract

**Introduction:**

Chinese herbal medicines (CHM) have been commonly used in the treatment of primary dysmenorrhea in East Asia. Several systematic reviews have been conducted to assess the clinical efficacy of CHM in the treatment of primary dysmenorrhea. However, their comparative efficacy is still unclear. Therefore, the purpose of this study is to conduct a network meta-analysis (NMA) to systematically compare the advantages of different CHM in the treatment of primary dysmenorrhea.

**Methods and analysis:**

The following electronic databases will be searched in this study: Web of Science, PubMed, Cochrane Library, Chinese Biomedical Literature Database, Chinese National Knowledge Infrastructure, Chinese Scientific Journal Database, and Wan-fang Database. Search terms include (Chinese herbal medicine or Chinese patent medicine or medicinal plants or phytotherapy or traditional medicine or Chinese herbal drugs or plant extracts or herbal medicine or herbal extract or herb or traditional Chinese medicine) and (primary dysmenorrhea or dysmenorrhea or painful menstruation) and (randomized controlled trial). The language will be limited to Chinese and English, and the search date will be up to May 2019. The included studies must be randomized controlled trials (RCTs) with patients diagnosed with primary dysmenorrhea. CHM must be used as interventions in the experimental group. While in the control group, studies that used a different herbal medicine, nonsteroidal anti-inflammatory drugs (NSAIDs), or placebo will be included. The primary outcomes include clinical efficacy and visual analog scale (VAS), and the secondary outcomes include adverse events and quality of life. Four reviewers will independently extract the data and assess the qualities of the studies. Statistical analysis will be conducted with R package for each outcome.

**Ethics and dissemination:**

Ethical approval is not required as this NMA is based on published studies. The completed NMA will be published in a peer-reviewed scientific journal.

**Trial registration number:**

PROSPERO CRD42018095254.

**Key Points**

- It will be the first network meta-analysis of different Chinese herbal medicines in their comparative efficacy in the treatment of primary dysmenorrhea.
- The clinical efficacy of Chinese herbal medicines in improvement of menstrual pain will be comprehensively assessed.
- The comparison between some Chinese herbal medicines will be first conducted head to head with the application of the network meta-analysis.
- The major challenge may come from the risk bias of the included studies, as some Chinese studies may do not clearly report methods for randomization, allocation concealment, and blinding.

## 1 Introduction

Primary dysmenorrhea is a common periodic menstrual pain in young women without pelvic pathology.^[1]^ Females with primary dysmenorrhea may suffer the pain for several days, seriously affecting their life qualities.^[2]^ Nonsteroidal anti-inflammatory drugs (NSAIDs) or oral contraceptives are the most commonly used medicines in the treatment of primary dysmenorrhea.^[3]^ However, long-term use of these medicines has been reported to have side effects, such as nausea or stomach pain.^[4,5]^ This leads to many patients seek complementary and alternative medicine (CAM) to manage the pain.

Chinese herbal medicines (CHM) are well-accepted in the treatment of primary dysmenorrhea in East Asia, such as China, Korea, and Japan.^[6–8]^ Many meta-analyses have been conducted to assess the clinical efficacy of different CHM. Lee et al analyzed the efficacy of Danggui Shaoyao San (DSS) in the treatment of primary dysmenorrhea in a meta-analysis and concluded that DSS was superior to analgesics.^[9]^ Zhu et al included 3475 women in a systematic review to assess the efficacy and safety of traditional Chinese medicines in the treatment of primary dysmenorrhea, and results showed a significant advantage compared with other treatments.^[10]^ Similar conclusions were also obtained in the meta-analysis of Shaofu Zhuyu decoction (SZD),^[11]^ Wenjing decoction,^[12]^ and Xuefu Zhuyu decoction.^[13]^

Many researchers have conducted experiments on the mechanisms of CHM in the treatment of primary dysmenorrhea. Hsu et al analyzed the physiological mechanism of Wenjing decoction on uterine contractility in vitro and found that the antagonism of PGF_2α_ (prostaglandin F2alpha) and ACh (acetylcholine) was the major mechanism in the treatment of primary dysmenorrhea.^[14]^*Angelica sinensis* has active components such as ferulic acid, which shows an inhibitory effect on uterine movement.^[15]^ The evodiamine in tetradium ruticarpum has been recommended for abdominal pain and dysmenorrhea.^[16]^ Cinnamic acid and cinnamic aldehyde in *Cinnamomum cassia* Presl inhibit uterine contractions by reducing the PGF_2α_ level and intracellular Ca^++^ to suppress COX-2 (Cyclooxygenase-2) and OTR (oxytocin receptor) expression.^[17]^

There are so many CHM being used in the treatment of primary dysmenorrhea in clinical practice. However, their comparative efficacy is still unclear. Therefore, the purpose of this study is to conduct a network meta-analysis (NMA) to systematically compare the advantages of different CHM in the treatment of primary dysmenorrhea.

## 2 Methods and analysis

### 2.1 Study registration

The protocol of this meta-analysis has been registered in PROSPERO with the registration number CRD42018095254.

### 2.2 Database and search strategies

**Figure 1:**
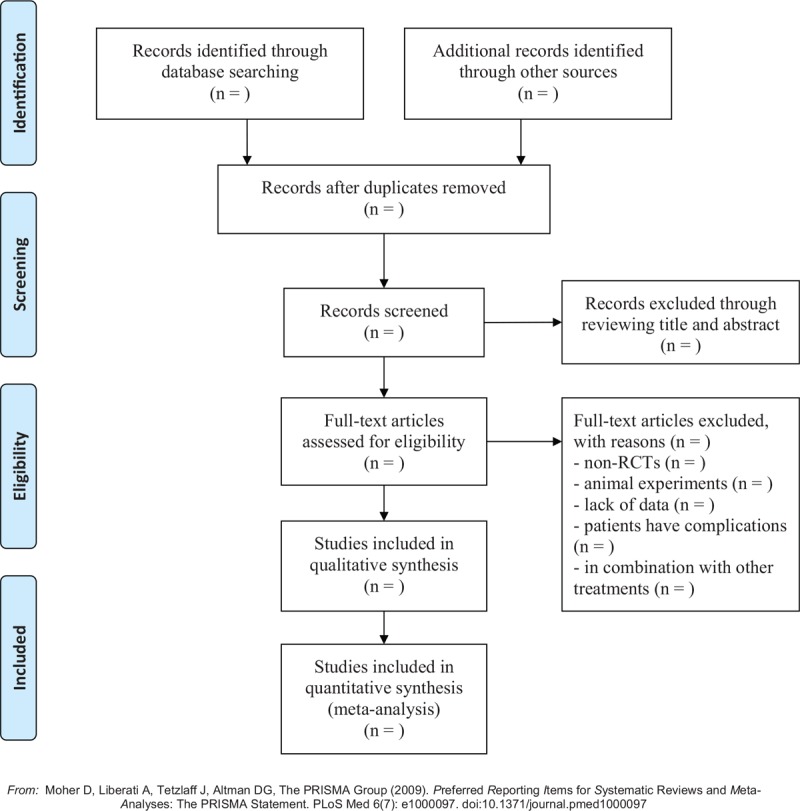
PRISMA flow diagram of the screening process.

The following electronic databases will be searched in this study: Web of Science, PubMed, Cochrane Library, Chinese Biomedical Literature Database, Chinese National Knowledge Infrastructure, Chinese Scientific Journal Database, and Wan-Fang Database. Search terms include (Chinese herbal medicine or Chinese patent medicine or medicinal plants or phytotherapy or traditional medicine or Chinese herbal drugs or plant extracts or herbal medicine or herbal extract or herb or traditional Chinese medicine) and (primary dysmenorrhea or dysmenorrhea or painful menstruation) and (randomized controlled trial). Additional eligible studies will be identified by hand searching relevant systematic reviews. The language will be limited to Chinese and English, and the search date will be up to May 2019. The screening process of the searched studies will be conducted according to the PRISMA flow diagram,^[18]^ as shown in Figure 1.

### 2.3 Inclusion criteria

#### 2.3.1 Patients

The included studies must be randomized controlled trials (RCTs) with patients diagnosed with primary dysmenorrhea. Primary dysmenorrhea is defined as a lower abdomen pain that occurs during menses. Patients that were diagnosed with pelvic pathology or secondary dysmenorrhea will be excluded.

#### 2.3.2 Interventions

In the experimental group, CHM must be used as the interventions for pain relief. CHM contain a very wide range of medicines, the most commonly used are DSS, SZD, Wenjing decoction, Xiaoyao San, and Xuefu Zhuyu decoction. For those not commonly used CHM, with the approval of all the reviewers, will also be included. In the control group, studies that used a different herbal medicine, NSAIDs, or placebo will be included.

### 2.4 Exclusion criteria

Studies that meet the exclusion criteria will be excluded:

(a) Non-RCTs, case studies, experience summaries, and animal experiments;
(b) Unpublished or repeated studies;
(c) Patients that were diagnosed with pregnancy, stroke, or with a severe drug allergic history in CHM;
(d) CHM in combination with acupuncture, massage, cupping, qigong, moxibustion, NSAIDs, or some other treatments, which should be approved by all the reviewers.

### 2.5 Outcomes

The primary outcomes include clinical efficacy and visual analog scale (VAS). In some Chinese studies, the clinical therapeutic effects are divided into 4 categories according to the self-reported symptoms of the patients after treatment. Those are cured (menstrual pain disappears, no recurrence after treatment); significant improved (significant improvements in menstrual pain, occasional recurrence after treatment), improved (improvements in menstrual pain, recurrence after treatment), and no effect (no improvements in menstrual pain or the symptoms have deteriorated). To conduct the analysis of dichotomous data, we define the clinical efficacy as 2 categories, 1 is the accumulation of cured, significant improved, and improved, and the other 1 is no effect.

VAS is a 100 mm long horizontal line with 2 endpoints marked as “no pain” and “worst pain imaginable”, and it is the most frequently used instrument to measure the pain scale of dysmenorrhea.^[19]^ Patients scored their pain before and after treatment, a decrease in the score means a reduction in pain.

The secondary outcomes are adverse events and quality of life. Symptoms often co-occurring with primary dysmenorrhea include diarrhea, skin rash, and nausea.^[1]^ Diarrhea refers to abnormal bowel movements. Skin rash refers to some changes in the skin traits. Nausea refers to an urge to vomit. Quality of life was measured using questionnaires before and after treatment.

### 2.6 Data extraction and quality assessment

Four reviewers (GL, XZ, JC, and WW) will independently extract the data and assess the qualities of the studies. Any disagreement will be resolved by discussions among all reviewers. Data of the included studies will be collected, including first author, language used, study design, sample sizes, diagnostic criteria, patients’ ages, duration of disease, interventions used in the experimental group and control group, treatment duration, outcomes, and adverse events. When the data of the study is insufficient, the original corresponding author of the study will be contacted to supplement the information. If it is unsuccessful, the study will be excluded. The risk of bias in each study will be assessed by 7 items using the Cochrane tool. For each item, 3 categories will be applied, including low, unclear, and high.^[20]^

### 2.7 Data analysis

Statistical analysis will be conducted with R package for each outcome. Relative risk, including 95% confidence intervals, will be used for analysis of dichotomous data on clinical efficacy. Mean difference will be used for analysis of continuous data on VAS. Before NMA, standard pairwise meta-analysis will be conducted for each formula. If the formula has many different forms, such as decoction, tablet, granule, or Chinese patent medicine, they will be classified as 1 group in the meta-analysis. Data will be extracted for all time points if sufficient data are available, and outcomes in different time points will be analyzed. NMA will be conducted to compare the clinical efficacy of different CHM directly or indirectly. The consistency will be assessed by node-splitting method, which estimates the direct and indirect treatment effect separately.^[21]^ If inconsistency is identified, potential sources of the inconsistency need to be detected.

### 2.8 Heterogeneity

Heterogeneity will be tested with the I^2^ statistic. In the meta-analysis, if I^2^ statistic is higher than 50%, which indicates there is high heterogeneity, a random model will be used;^[20]^ otherwise, a fixed model will be used. In the NMA, a total I^2^ statistic will be reported, and only random effects model will be used. If there is a high heterogeneity, potential sources need to be detected according to methods of sensitivity analysis and subgroup analysis. If the data is inappropriate to be pooled or subgroup analyzed, a narrative description will be conducted to provide an analysis in the findings of these studies. If sufficient data are available, subgroup analysis will be conducted to compare the following categories:

(1) age,
(2) intervention types of control group,
(3) duration or severity of primary dysmenorrhea;
(4) types of CHM.

### 2.9 Patient and public involvement

As this NMA is based on published studies, patient and the public are not involved in the study.

## 3 Ethics and dissemination

Currently, CHM has been widely used by Chinese physicians in the treatment of primary dysmenorrhea. Several meta-analyses have been conducted to assess the clinical efficacy of different CHM. However, the comparative efficacy of different CHM is still unclear. Therefore, the purpose of this study is to systematically compare the advantages of different CHM in the treatment of primary dysmenorrhea in the NMA. This NMA will provide comprehensive insights in CHM in the treatment of primary dysmenorrhea, which will benefit the medical physicians who may use CHM in the treatments.

Ethical approval is not required as this NMA is based on published studies. The completed NMA will be published in a peer-reviewed scientific journal.

## Author contributions

Gao L, Jia C, and Wang W conceived the study and participated in the design of search strategy. Gao L, Xiao Z, and Jia C drafted the manuscript. Xiao Z and Wang W revised the manuscript. All authors approved the final manuscript to submit for publication.

**Conceptualization:** Li Gao, Chunhua Jia, Wei Wang.

**Writing – original draft:** Li Gao, Zhuoran Xiao, Chunhua Jia.

**Writing – review & editing:** Zhuoran Xiao, Wei Wang.
